# Dr. Mahendra T. Mehta 1929–2008

**Published:** 2008

**Authors:** Pravin Kanabar

**Affiliations:** *Past President, Indian Orthopaedic Association, Opp. Hasubhai Chamber, Behind Townhall Ellis Bridge, Ahmedabad - 380 006, India. E-mail: drkanabar@googlemail.com*

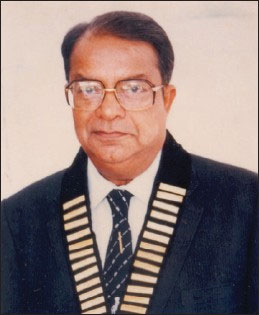



Dr. Mahendra T. Mehta was the first Orthopedic Surgeon of Gujarat and the President of the Indian Orthopedic Association from 1988 to 1989. He was born in Zariya, Bihar on February 5^th^, 1929. He studied at Carmichael Medical College, Kolkata, and completed his M.B.B.S. in 1952. He joined Patna University in 1954 for a postgraduate degree in General Surgery and graduated with an M.S. (Ortho) degree from Patna in 1957.

His first research project, 'Diaphysecomy and Bone Regeneration' initiated in Patna in 1955, was supported by a scholarship from Lady Tata Memorial Trust. He joined Sheth Vadilal Sarabhai Hospital in 1957 and later in 1961, joined Civil Hospital, Ahmedabad as an Honorary Orthopedic Surgeon. He continued his research here with a research grant from Gujarat University, and finally presented his work at the SICOT meeting in Copenhagen in 1975.

He had a great interest in C.T.E.V and considered manual correction and maintenance with a plaster cast to be a mainstay in its management. Armed with the training imparted by his mentor, Dr. B. Mukhopadyay, he started anterolateral decompression for Pott's Paraplegia in 1957 in Ahmedabad. In the late 1950s, he gave up immobilization and accepted active mobilization for tuberculosis of joints. He was awarded the B. N. Mehta Oration Award in 1974 after his presentation of his work on bone and joint tuberculosis.

He was a teacher to hundreds of students, yet his humble words to his family in the last week of his life were, 'I am a student and I have yet to learn a lot.' Not only was he a host for many medical students, doctors, and visiting orthopedic surgeon friends, but he also looked after all Johnson and Johnson fellows during their visits to Ahmedabad. Family members concede: 'He taught us to transform relations to dear ones and dear ones to great relationships'. His family has lost a most dear one, but feels his presence all the time, even after his death.

He offered deep insights into orthopedics as a member of the Advisory committee appointed by the IOA in 1980 for the standardization of a postgraduate (PG) degree in orthopedics in India. The committee brought in the idea of a PG work book, a precursor of the log book. He served B. J. Medical College and Civil Hospital as an Honorary Professor of Orthopedics from 1961 to 1981, and served various hospitals in Ahmedabad, including the Cancer Hospital in Ahmedabad.

He was a recipient of P. R. Trivedi (1980) and Dr. Katrak (1982) Oration Awards. He was the founder President of the Gujarat Orthopedic Association. He published, 'Forty years of Orthopedics—As I Saw It' in February 1998. It's a small book, but a treasure of precious thoughts.

He has made a great difference for many lives, not only as an orthopedic surgeon, but also as a great human being. In his death on August 14, 2008, Yasubhabhi has lost a partner who greatly loved her and always took her with him on all his travels. In his death, we orthopedic surgeons in Gujarat have lost a father, who kept all of us together in our nest.

May God Almighty rest his soul in Eternal Peace.

